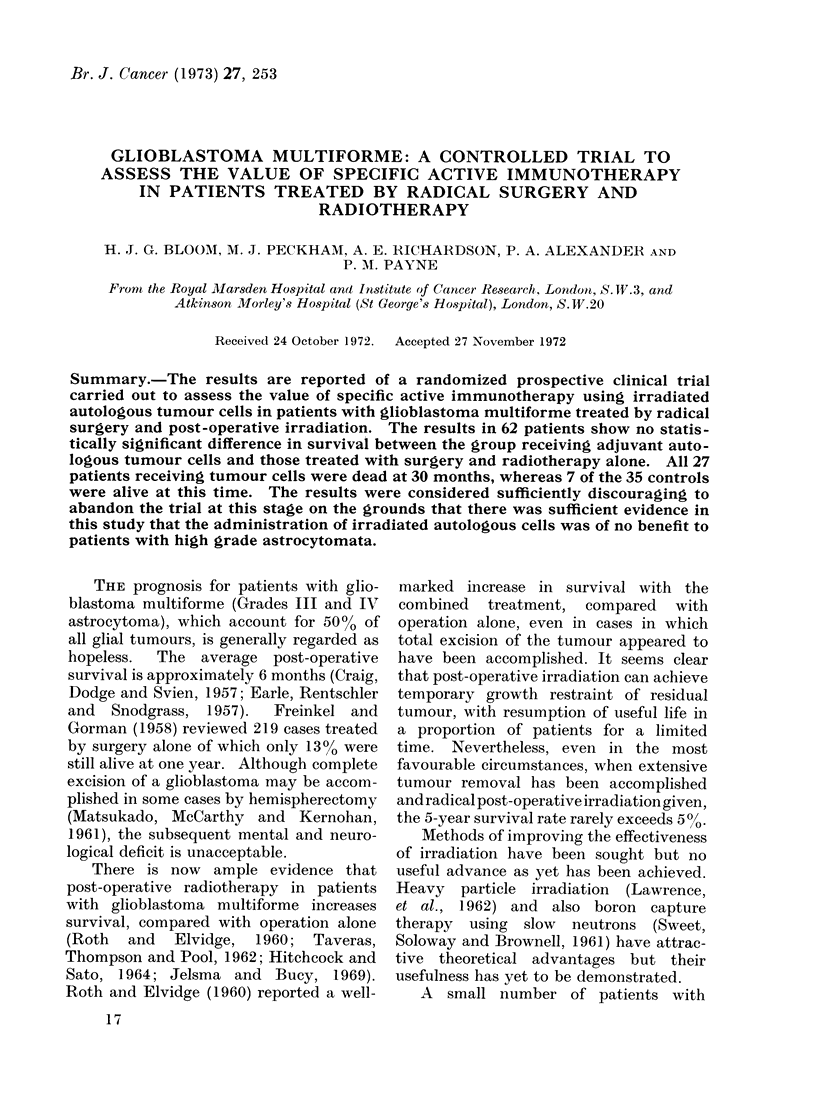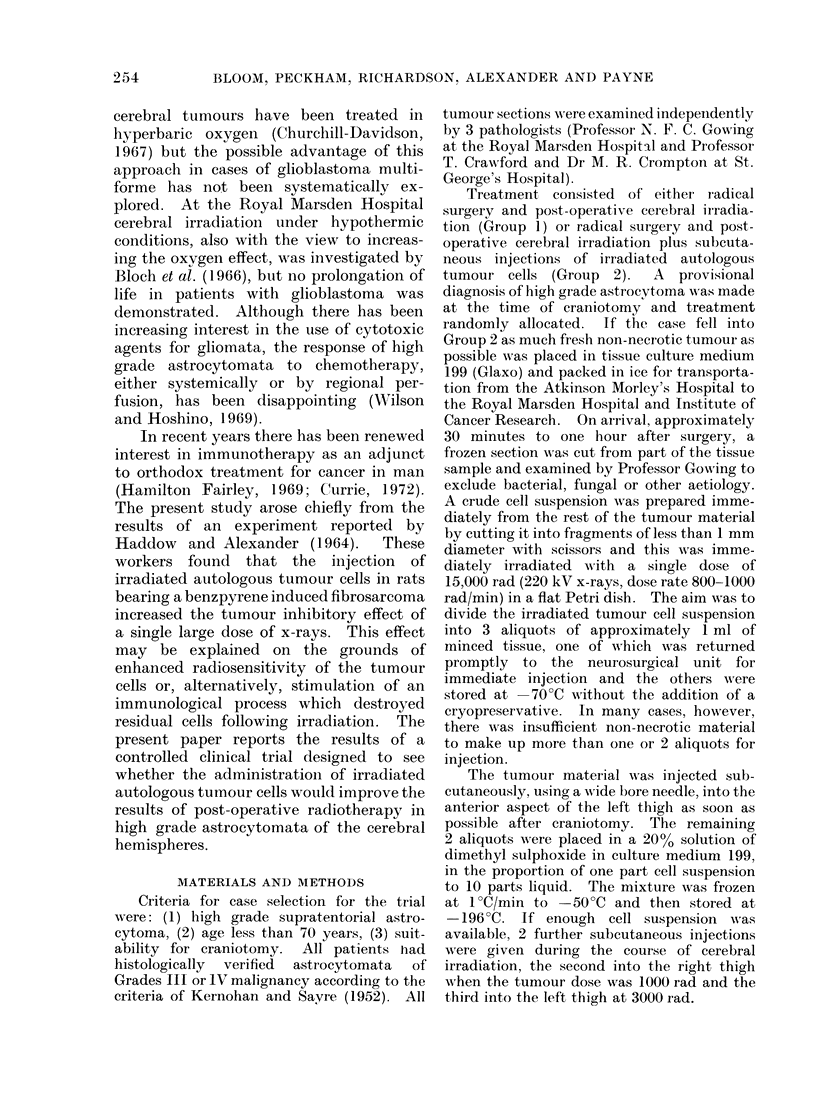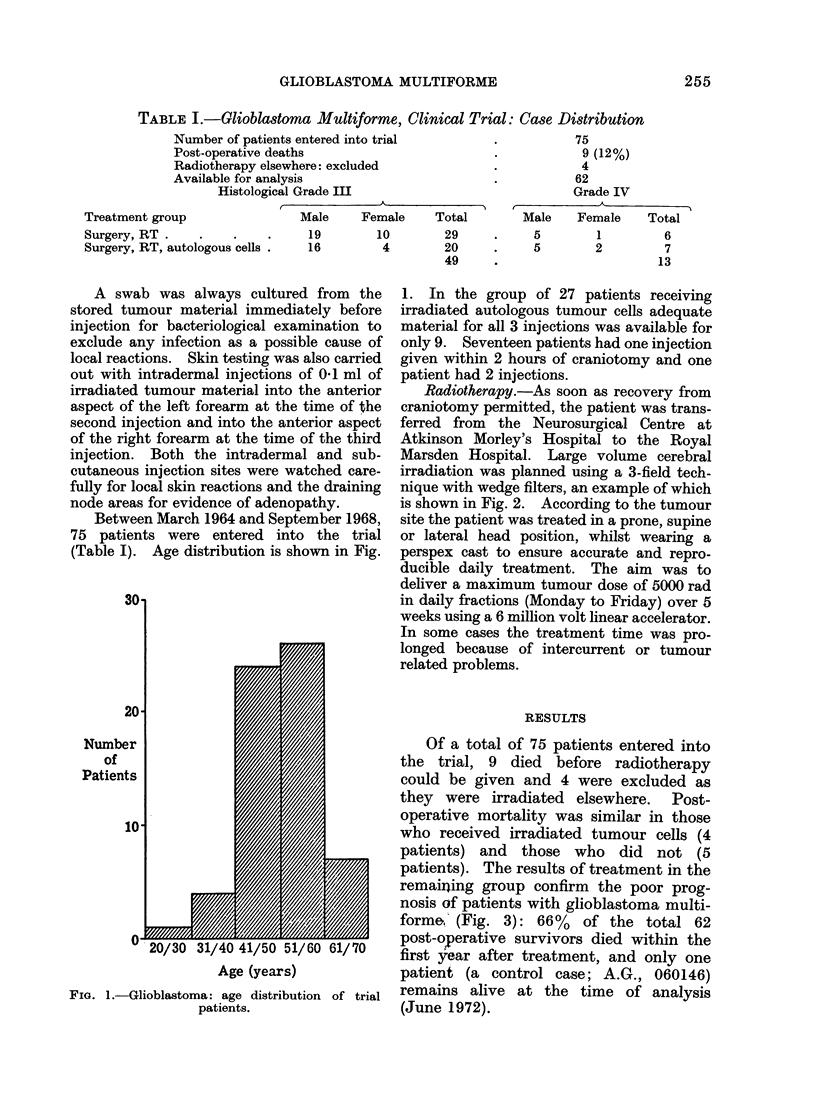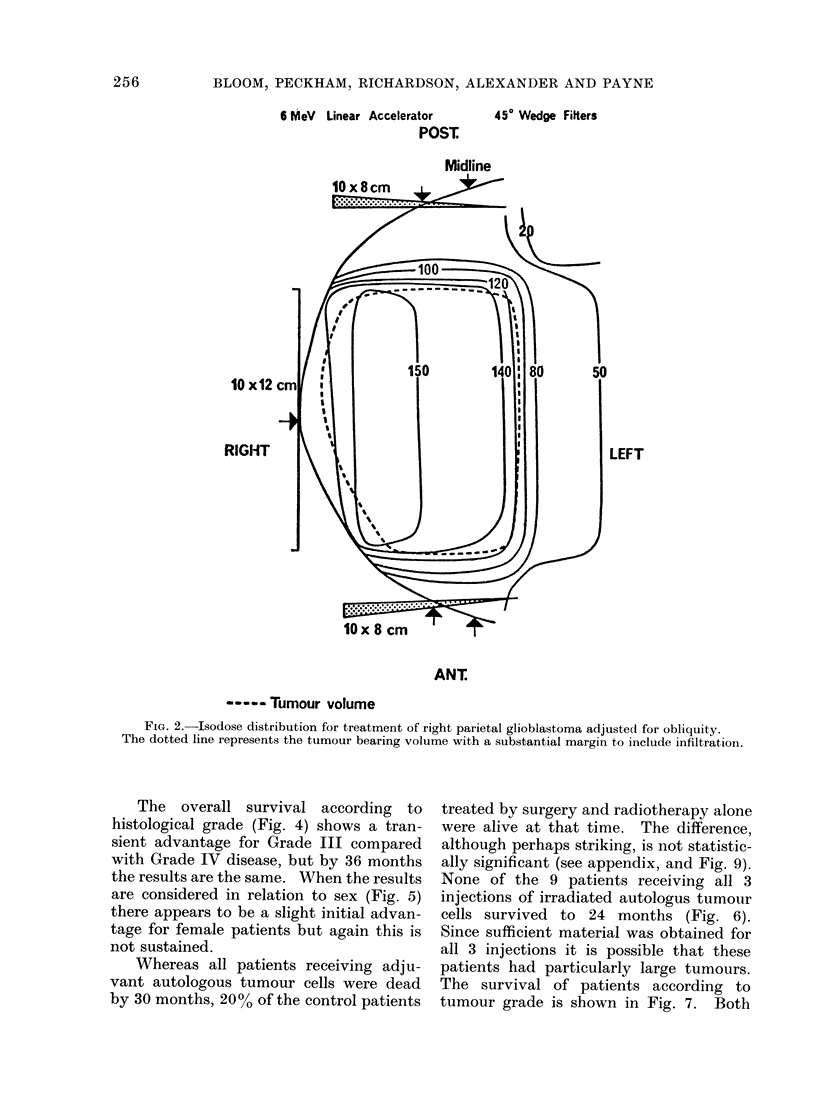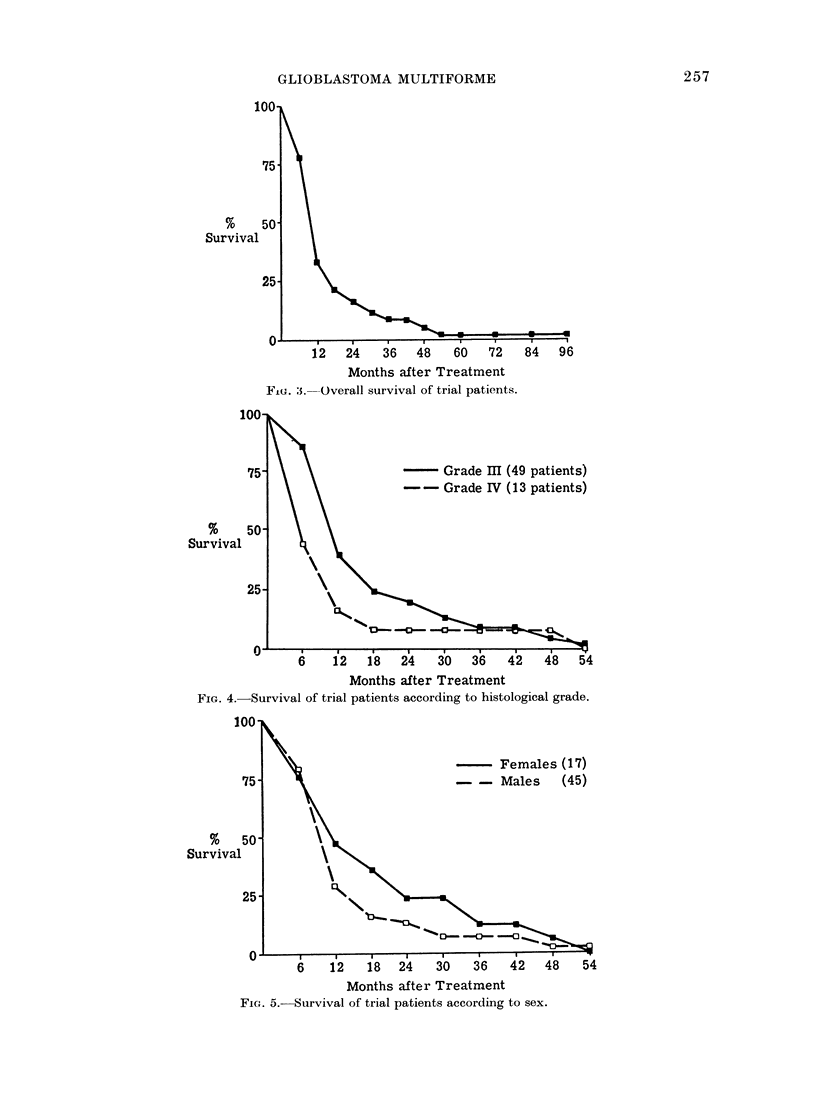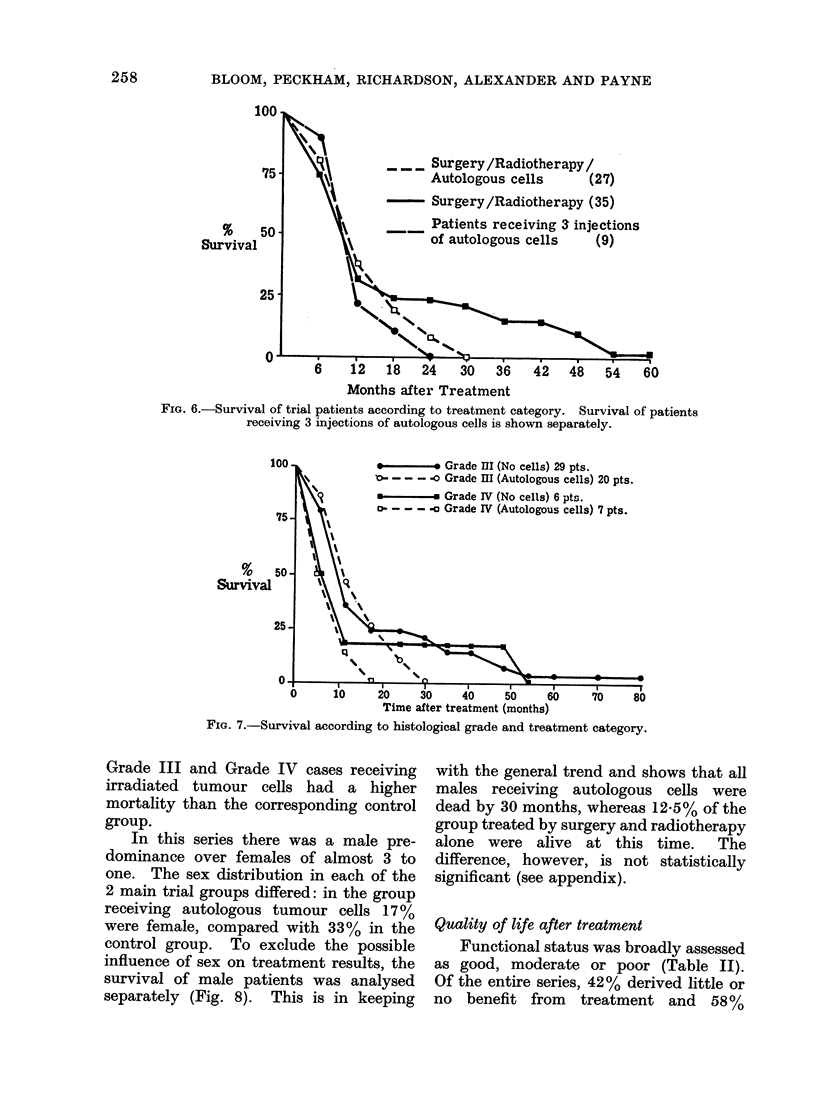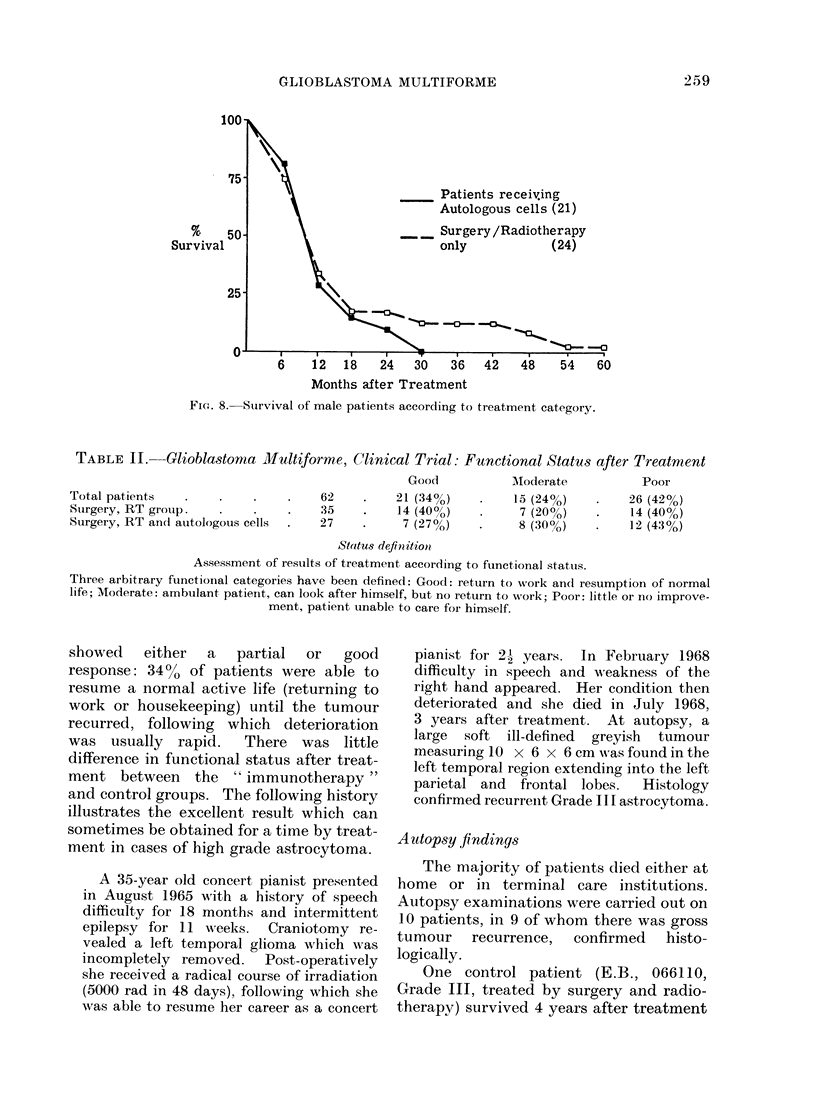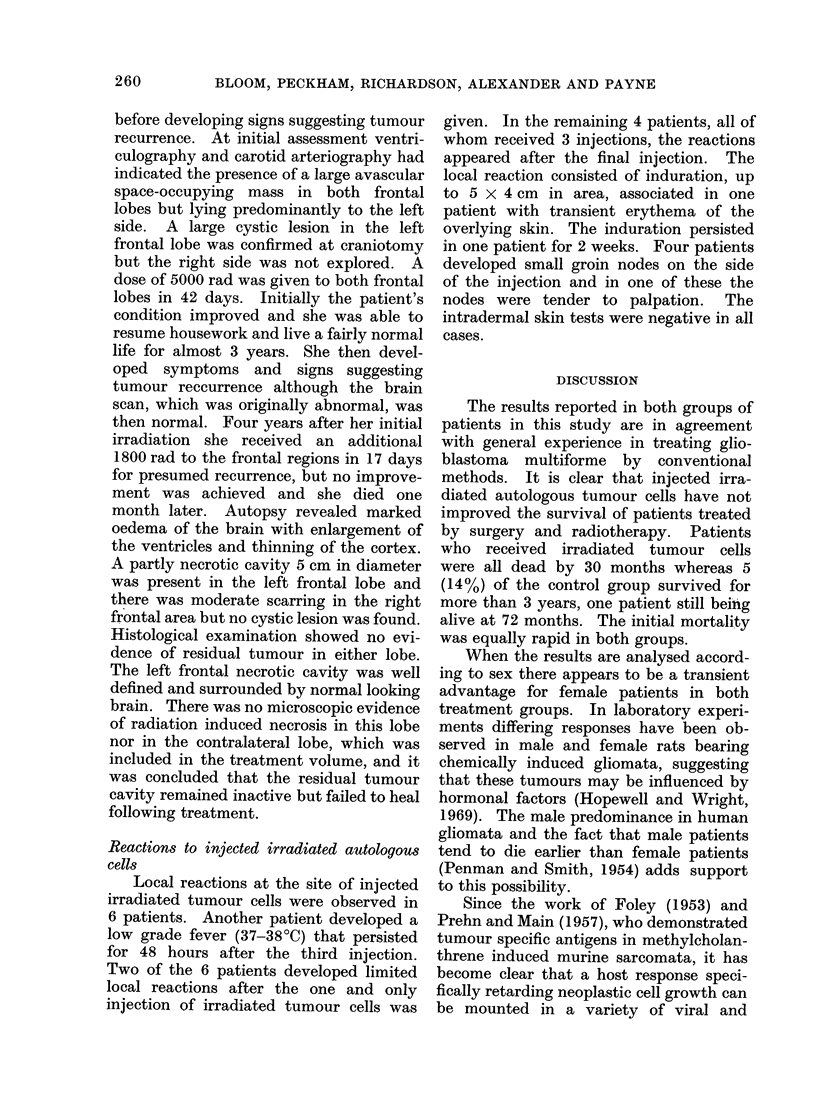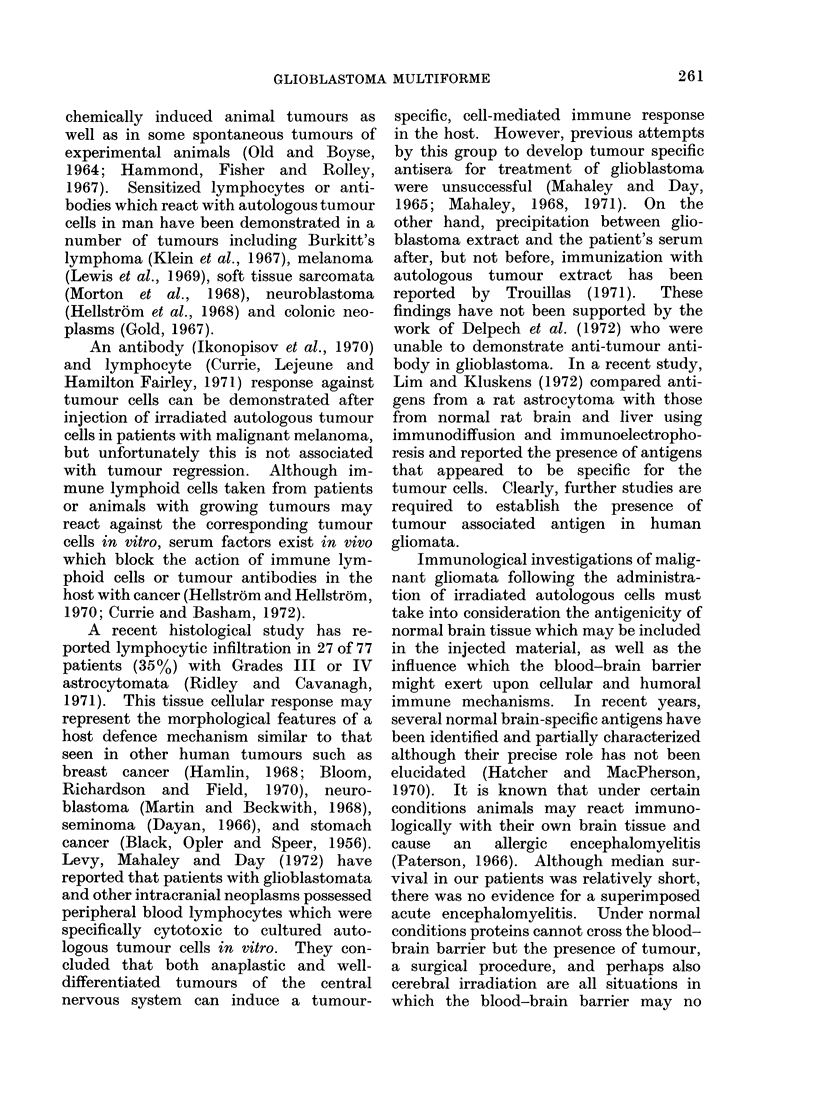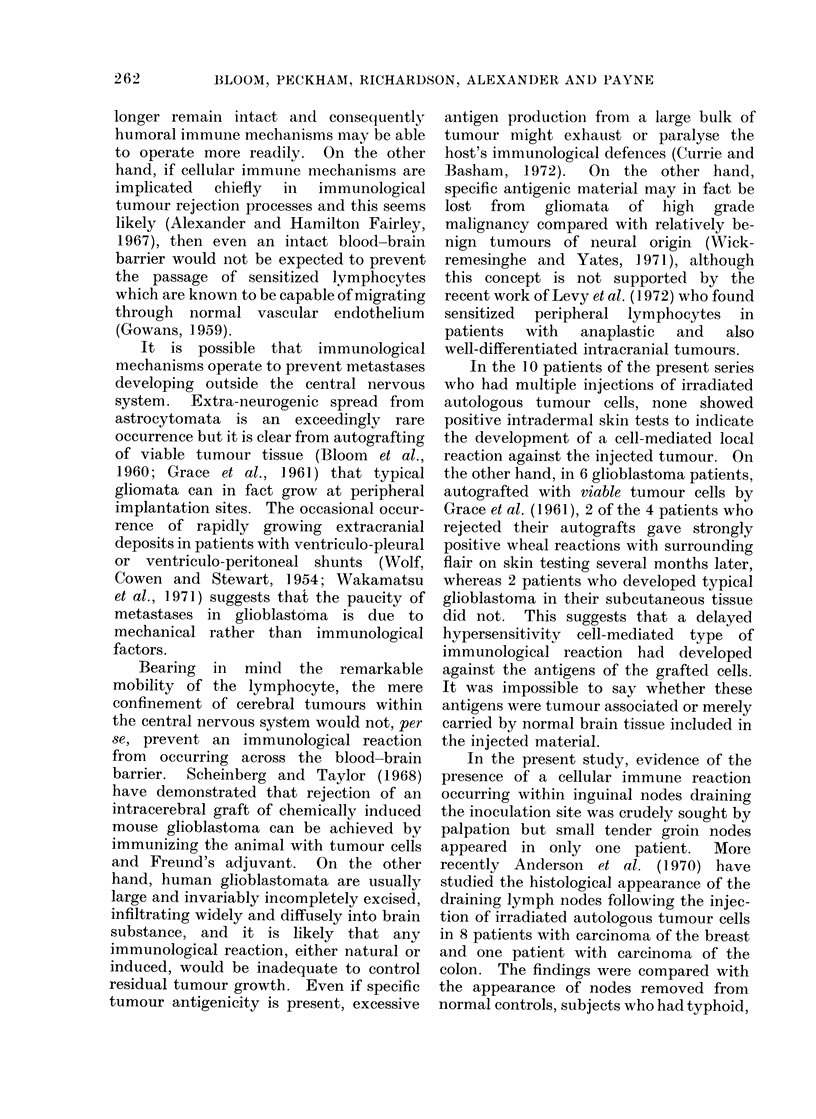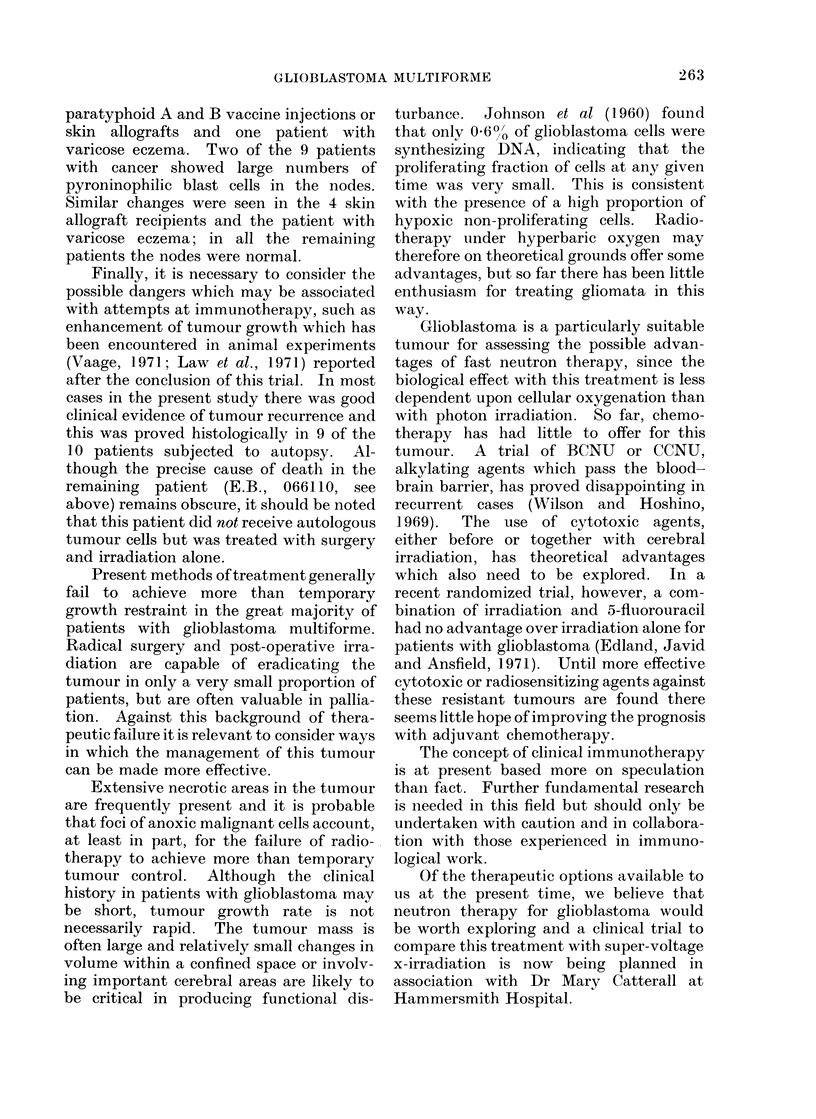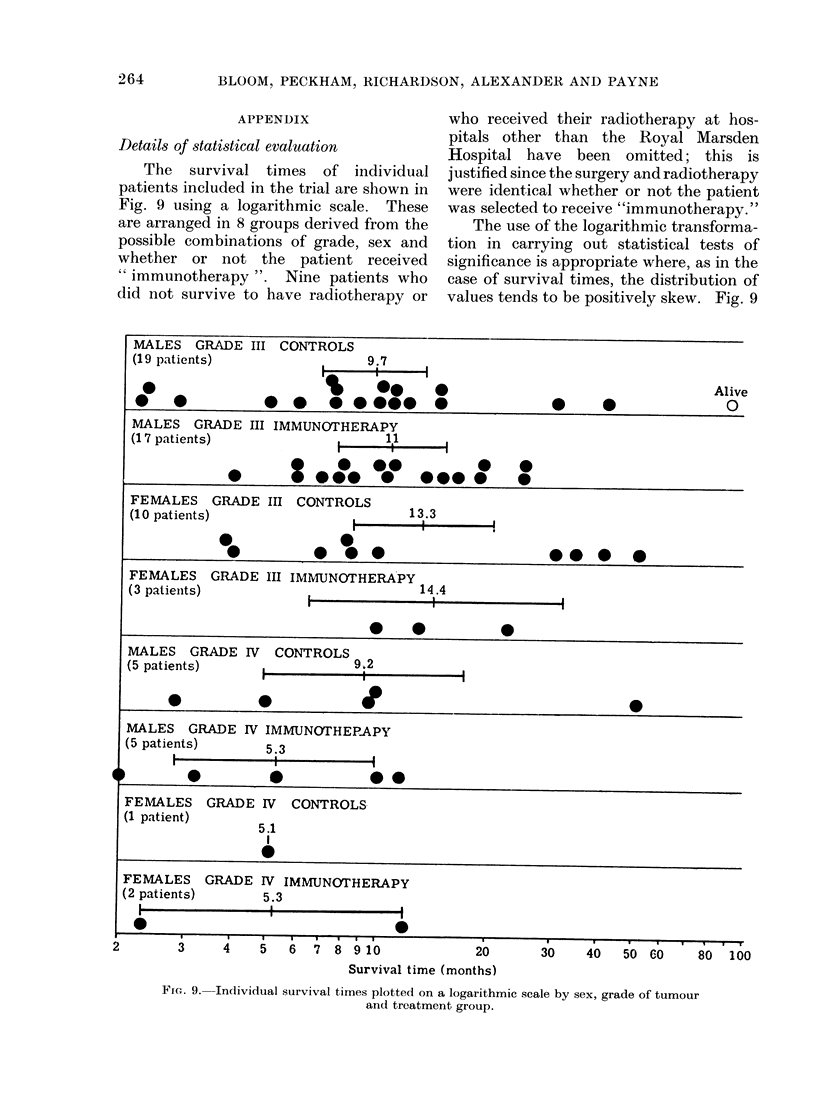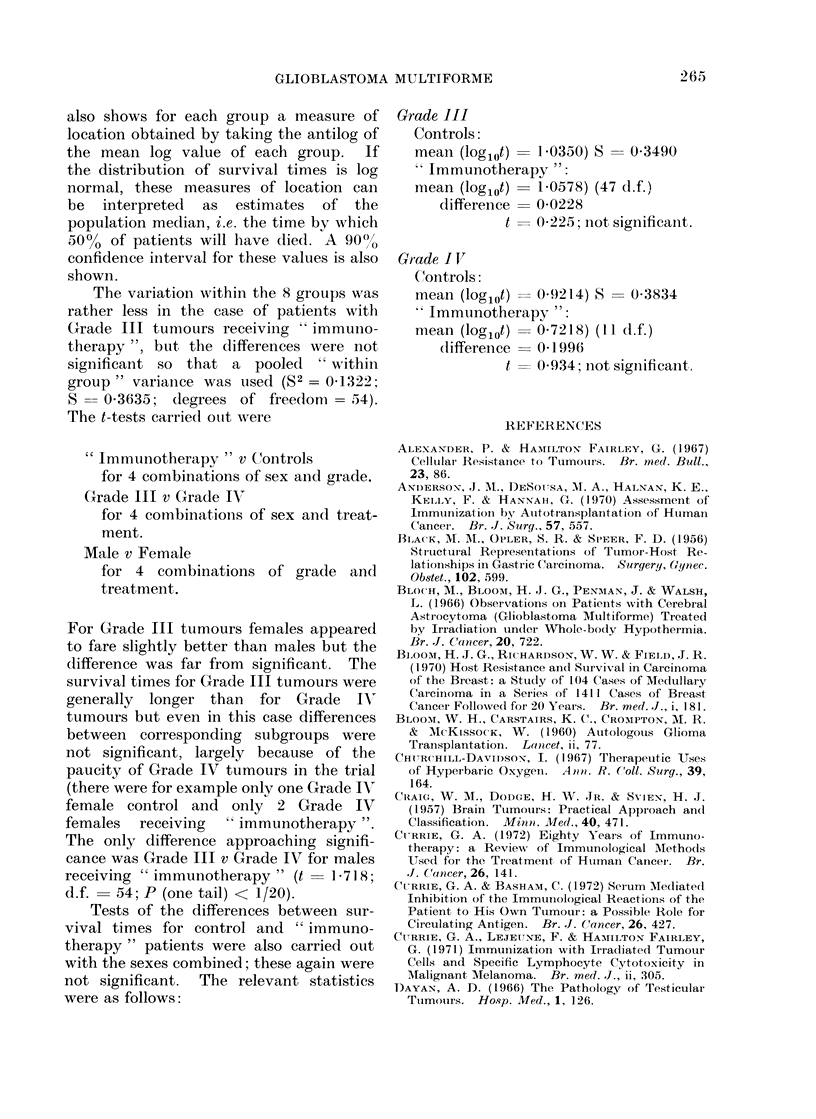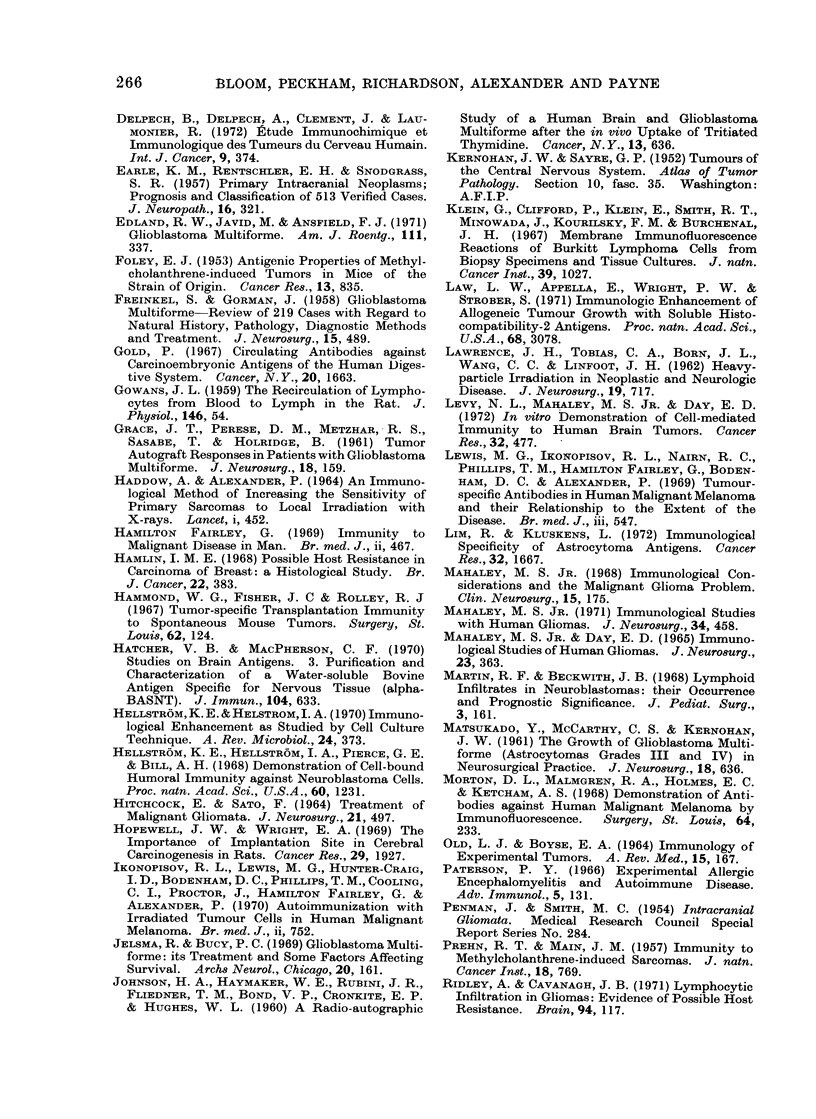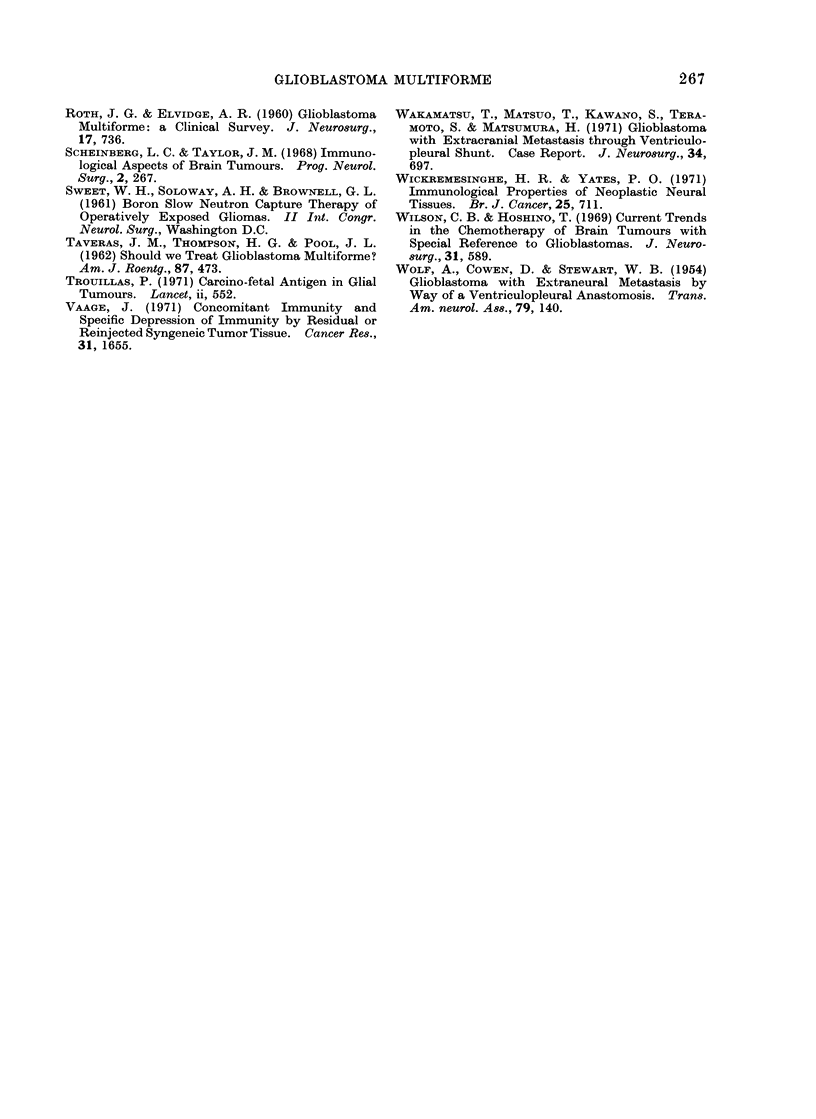# Glioblastoma Multiforme: A Controlled Trial to Assess the Value of Specific Active Immunotherapy in Patients Treated by Radical Surgery and Radiotherapy

**DOI:** 10.1038/bjc.1973.30

**Published:** 1973-03

**Authors:** H. J. G. Bloom, M. J. Peckham, A. E. Richardson, P. A. Alexander, P. M. Payne

## Abstract

The results are reported of a randomized prospective clinical trial carried out to assess the value of specific active immunotherapy using irradiated autologous tumour cells in patients with glioblastoma multiforme treated by radical surgery and post-operative irradiation. The results in 62 patients show no statistically significant difference in survival between the group receiving adjuvant autologous tumour cells and those treated with surgery and radiotherapy alone. All 27 patients receiving tumour cells were dead at 30 months, whereas 7 of the 35 controls were alive at this time. The results were considered sufficiently discouraging to abandon the trial at this stage on the grounds that there was sufficient evidence in this study that the administration of irradiated autologous cells was of no benefit to patients with high grade astrocytomata.


*Br. J. Cancer* (1973) **27**, 253

### GLIOBLASTOMA MULTIFORME: A CONTROLLED TRIAL TO ASSESS THE VALUE OF SPECIFIC ACTIVE IMMUNOTHERAPY IN PATIENTS TREATED BY RADICAL SURGERY AND RADIOTHERAPY

H. J. G. BLOOM, M. J. PECKHAM, A. E. RICHARDSON, P. A. ALEXANDER AND P. M. PAYNE

*From the Royal Marsden Hospital and Institute of Cancer Research, London, S.W.3, and Atkinson Morley's Hospital (St George's Hospital), London, S.W.20*

Received 24 October 1972. Accepted 27 November 1972

**Summary.—The results are reported of a randomized prospective clinical trial carried out to assess the value of specific active immunotherapy using irradiated autologous tumour cells in patients with glioblastoma multiforme treated by radical surgery and post-operative irradiation. The results in 62 patients show no statistically significant difference in survival between the group receiving adjuvant autologous tumour cells and those treated with surgery and radiotherapy alone. All 27 patients receiving tumour cells were dead at 30 months, whereas 7 of the 35 controls were alive at this time. The results were considered sufficiently discouraging to abandon the trial at this stage on the grounds that there was sufficient evidence in this study that the administration of irradiated autologous cells was of no benefit to patients with high grade astrocytomata.**

THE prognosis for patients with glioblastoma multiforme (Grades III and IV astrocytoma), which account for 50% of all glial tumours, is generally regarded as hopeless. The average post-operative survival is approximately 6 months (Craig, Dodge and Svien, 1957; Earle, Rentschler and Snodgrass, 1957). Freinkel and Gorman (1958) reviewed 219 cases treated by surgery alone of which only 13% were still alive at one year. Although complete excision of a glioblastoma may be accomplished in some cases by hemispherectomy (Matsukado, McCarthy and Kernohan, 1961), the subsequent mental and neurological deficit is unacceptable.

There is now ample evidence that post-operative radiotherapy in patients with glioblastoma multiforme increases survival, compared with operation alone (Roth and Elvidge, 1960; Taveras, Thompson and Pool, 1962; Hitchcock and Sato, 1964; Jelsma and Bucy, 1969). Roth and Elvidge (1960) reported a well-marked increase in survival with the combined treatment, compared with operation alone, even in cases in which total excision of the tumour appeared to have been accomplished. It seems clear that post-operative irradiation can achieve temporary growth restraint of residual tumour, with resumption of useful life in a proportion of patients for a limited time. Nevertheless, even in the most favourable circumstances, when extensive tumour removal has been accomplished and radical post-operative irradiation given, the 5-year survival rate rarely exceeds 5%.

Methods of improving the effectiveness of irradiation have been sought but no useful advance as yet has been achieved. Heavy particle irradiation (Lawrence, *et al.*, 1962) and also boron capture therapy using slow neutrons (Sweet, Soloway and Brownell, 1961) have attractive theoretical advantages but their usefulness has yet to be demonstrated.

A small number of patients with cerebral tumours have been treated in hyperbaric oxygen (Churchill-Davidson, 1967) but the possible advantage of this approach in cases of glioblastoma multiforme has not been systematically explored. At the Royal Marsden Hospital cerebral irradiation under hypothermic conditions, also with the view to increasing the oxygen effect, was investigated by Bloch *et al.* (1966), but no prolongation of life in patients with glioblastoma was demonstrated. Although there has been increasing interest in the use of cytotoxic agents for gliomata, the response of high grade astrocytomata to chemotherapy, either systemically or by regional perfusion, has been disappointing (Wilson and Hoshino, 1969).

In recent years there has been renewed interest in immunotherapy as an adjunct to orthodox treatment for cancer in man (Hamilton Fairley, 1969; Currie, 1972). The present study arose chiefly from the results of an experiment reported by Haddow and Alexander (1964). These workers found that the injection of irradiated autologous tumour cells in rats bearing a benzpyrene induced fibrosarcoma increased the tumour inhibitory effect of a single large dose of x-rays. This effect may be explained on the grounds of enhanced radiosensitivity of the tumour cells or, alternatively, stimulation of an immunological process which destroyed residual cells following irradiation. The present paper reports the results of a controlled clinical trial designed to see whether the administration of irradiated autologous tumour cells would improve the results of post-operative radiotherapy in high grade astrocytomata of the cerebral hemispheres.

### MATERIALS AND METHODS

Criteria for case selection for the trial were: (1) high grade supratentorial astrocytoma, (2) age less than 70 years, (3) suitability for craniotomy. All patients had histologically verified astrocytomata of Grades III or IV malignancy according to the criteria of Kernohan and Sayre (1952). All tumour sections were examined independently by 3 pathologists (Professor N. F. C. Gowing at the Royal Marsden Hospital and Professor T. Crawford and Dr M. R. Crompton at St. George's Hospital).

Treatment consisted of either radical surgery and post-operative cerebral irradiation (Group 1) or radical surgery and post-operative cerebral irradiation plus subcutaneous injections of irradiated autologous tumour cells (Group 2). A provisional diagnosis of high grade astrocytoma was made at the time of craniotomy and treatment randomly allocated. If the case fell into Group 2 as much fresh non-necrotic tumour as possible was placed in tissue culture medium 199 (Glaxo) and packed in ice for transportation from the Atkinson Morley's Hospital to the Royal Marsden Hospital and Institute of Cancer Research. On arrival, approximately 30 minutes to one hour after surgery, a frozen section was cut from part of the tissue sample and examined by Professor Gowing to exclude bacterial, fungal or other aetiology. A crude cell suspension was prepared immediately from the rest of the tumour material by cutting it into fragments of less than 1 mm diameter with scissors and this was immediately irradiated with a single dose of 15,000 rad (220 kV x-rays, dose rate 800–1000 rad/min) in a flat Petri dish. The aim was to divide the irradiated tumour cell suspension into 3 aliquots of approximately 1 ml of minced tissue, one of which was returned promptly to the neurosurgical unit for immediate injection and the others were stored at −70°C without the addition of a cryopreservative. In many cases, however, there was insufficient non-necrotic material to make up more than one or 2 aliquots for injection.

The tumour material was injected subcutaneously, using a wide bore needle, into the anterior aspect of the left thigh as soon as possible after craniotomy. The remaining 2 aliquots were placed in a 20% solution of dimethyl sulphoxide in culture medium 199, in the proportion of one part cell suspension to 10 parts liquid. The mixture was frozen at 1°C/min to −50°C and then stored at −196°C. If enough cell suspension was available, 2 further subcutaneous injections were given during the course of cerebral irradiation, the second into the right thigh when the tumour dose was 1000 rad and the third into the left thigh at 3000 rad.

TABLE I.—*Glioblastoma Multiforme, Clinical Trial: Case Distribution*

| | |
|---|---|
| Number of patients entered into trial | 75 |
| Post-operative deaths | 9 (12%) |
| Radiotherapy elsewhere: excluded | 4 |
| Available for analysis | 62 |

| Treatment group | Histological Grade III | | | Grade IV | | |
|---|---|---|---|---|---|---|
| | Male | Female | Total | Male | Female | Total |
| Surgery, RT | 19 | 10 | 29 | 5 | 1 | 6 |
| Surgery, RT, autologous cells | 16 | 4 | 20 | 5 | 2 | 7 |
| | | | 49 | | | 13 |

A swab was always cultured from the stored tumour material immediately before injection for bacteriological examination to exclude any infection as a possible cause of local reactions. Skin testing was also carried out with intradermal injections of 0·1 ml of irradiated tumour material into the anterior aspect of the left forearm at the time of the second injection and into the anterior aspect of the right forearm at the time of the third injection. Both the intradermal and subcutaneous injection sites were watched carefully for local skin reactions and the draining node areas for evidence of adenopathy.

Between March 1964 and September 1968, 75 patients were entered into the trial (Table I). Age distribution is shown in Fig. 1. In the group of 27 patients receiving irradiated autologous tumour cells adequate material for all 3 injections was available for only 9. Seventeen patients had one injection given within 2 hours of craniotomy and one patient had 2 injections.

FIG. 1.—Glioblastoma: age distribution of trial patients.

*Radiotherapy.*—As soon as recovery from craniotomy permitted, the patient was transferred from the Neurosurgical Centre at Atkinson Morley's Hospital to the Royal Marsden Hospital. Large volume cerebral irradiation was planned using a 3-field technique with wedge filters, an example of which is shown in Fig. 2. According to the tumour site the patient was treated in a prone, supine or lateral head position, whilst wearing a perspex cast to ensure accurate and reproducible daily treatment. The aim was to deliver a maximum tumour dose of 5000 rad in daily fractions (Monday to Friday) over 5 weeks using a 6 million volt linear accelerator. In some cases the treatment time was prolonged because of intercurrent or tumour related problems.

### RESULTS

Of a total of 75 patients entered into the trial, 9 died before radiotherapy could be given and 4 were excluded as they were irradiated elsewhere. Post-operative mortality was similar in those who received irradiated tumour cells (4 patients) and those who did not (5 patients). The results of treatment in the remaining group confirm the poor prognosis of patients with glioblastoma multiforme (Fig. 3): 66% of the total 62 post-operative survivors died within the first year after treatment, and only one patient (a control case; A.G., 060146) remains alive at the time of analysis (June 1972).

FIG. 2.—Isodose distribution for treatment of right parietal glioblastoma adjusted for obliquity. The dotted line represents the tumour bearing volume with a substantial margin to include infiltration.

The overall survival according to histological grade (Fig. 4) shows a transient advantage for Grade III compared with Grade IV disease, but by 36 months the results are the same. When the results are considered in relation to sex (Fig. 5) there appears to be a slight initial advantage for female patients but again this is not sustained.

Whereas all patients receiving adjuvant autologous tumour cells were dead by 30 months, 20% of the control patients treated by surgery and radiotherapy alone were alive at that time. The difference, although perhaps striking, is not statistically significant (see appendix, and Fig. 9). None of the 9 patients receiving all 3 injections of irradiated autologus tumour cells survived to 24 months (Fig. 6). Since sufficient material was obtained for all 3 injections it is possible that these patients had particularly large tumours. The survival of patients according to tumour grade is shown in Fig. 7. Both

Fig. 3.—Overall survival of trial patients.

Fig. 4.—Survival of trial patients according to histological grade.

Fig. 5.—Survival of trial patients according to sex.

FIG. 6.—Survival of trial patients according to treatment category. Survival of patients receiving 3 injections of autologous cells is shown separately.

FIG. 7.—Survival according to histological grade and treatment category.

Grade III and Grade IV cases receiving irradiated tumour cells had a higher mortality than the corresponding control group.

In this series there was a male predominance over females of almost 3 to one. The sex distribution in each of the 2 main trial groups differed: in the group receiving autologous tumour cells 17% were female, compared with 33% in the control group. To exclude the possible influence of sex on treatment results, the survival of male patients was analysed separately (Fig. 8). This is in keeping with the general trend and shows that all males receiving autologous cells were dead by 30 months, whereas 12·5% of the group treated by surgery and radiotherapy alone were alive at this time. The difference, however, is not statistically significant (see appendix).

### *Quality of life after treatment*

Functional status was broadly assessed as good, moderate or poor (Table II). Of the entire series, 42% derived little or no benefit from treatment and 58%

FIG. 8.—Survival of male patients according to treatment category.

TABLE II.—*Glioblastoma Multiforme, Clinical Trial: Functional Status after Treatment*

| | | Good | Moderate | Poor |
|---|---|---|---|---|
| Total patients | 62 | 21 (34%) | 15 (24%) | 26 (42%) |
| Surgery, RT group | 35 | 14 (40%) | 7 (20%) | 14 (40%) |
| Surgery, RT and autologous cells | 27 | 7 (27%) | 8 (30%) | 12 (43%) |

*Status definition*

Assessment of results of treatment according to functional status.

Three arbitrary functional categories have been defined: Good: return to work and resumption of normal life; Moderate: ambulant patient, can look after himself, but no return to work; Poor: little or no improvement, patient unable to care for himself.

showed either a partial or good response: 34% of patients were able to resume a normal active life (returning to work or housekeeping) until the tumour recurred, following which deterioration was usually rapid. There was little difference in functional status after treatment between the " immunotherapy " and control groups. The following history illustrates the excellent result which can sometimes be obtained for a time by treatment in cases of high grade astrocytoma.

A 35-year old concert pianist presented in August 1965 with a history of speech difficulty for 18 months and intermittent epilepsy for 11 weeks. Craniotomy revealed a left temporal glioma which was incompletely removed. Post-operatively she received a radical course of irradiation (5000 rad in 48 days), following which she was able to resume her career as a concert pianist for 2½ years. In February 1968 difficulty in speech and weakness of the right hand appeared. Her condition then deteriorated and she died in July 1968, 3 years after treatment. At autopsy, a large soft ill-defined greyish tumour measuring 10 × 6 × 6 cm was found in the left temporal region extending into the left parietal and frontal lobes. Histology confirmed recurrent Grade III astrocytoma.

*Autopsy findings*

The majority of patients died either at home or in terminal care institutions. Autopsy examinations were carried out on 10 patients, in 9 of whom there was gross tumour recurrence, confirmed histologically.

One control patient (E.B., 066110, Grade III, treated by surgery and radiotherapy) survived 4 years after treatment before developing signs suggesting tumour recurrence. At initial assessment ventriculography and carotid arteriography had indicated the presence of a large avascular space-occupying mass in both frontal lobes but lying predominantly to the left side. A large cystic lesion in the left frontal lobe was confirmed at craniotomy but the right side was not explored. A dose of 5000 rad was given to both frontal lobes in 42 days. Initially the patient's condition improved and she was able to resume housework and live a fairly normal life for almost 3 years. She then developed symptoms and signs suggesting tumour reccurrence although the brain scan, which was originally abnormal, was then normal. Four years after her initial irradiation she received an additional 1800 rad to the frontal regions in 17 days for presumed recurrence, but no improvement was achieved and she died one month later. Autopsy revealed marked oedema of the brain with enlargement of the ventricles and thinning of the cortex. A partly necrotic cavity 5 cm in diameter was present in the left frontal lobe and there was moderate scarring in the right frontal area but no cystic lesion was found. Histological examination showed no evidence of residual tumour in either lobe. The left frontal necrotic cavity was well defined and surrounded by normal looking brain. There was no microscopic evidence of radiation induced necrosis in this lobe nor in the contralateral lobe, which was included in the treatment volume, and it was concluded that the residual tumour cavity remained inactive but failed to heal following treatment.

*Reactions to injected irradiated autologous cells*

Local reactions at the site of injected irradiated tumour cells were observed in 6 patients. Another patient developed a low grade fever (37–38°C) that persisted for 48 hours after the third injection. Two of the 6 patients developed limited local reactions after the one and only injection of irradiated tumour cells was given. In the remaining 4 patients, all of whom received 3 injections, the reactions appeared after the final injection. The local reaction consisted of induration, up to $5 \times 4$ cm in area, associated in one patient with transient erythema of the overlying skin. The induration persisted in one patient for 2 weeks. Four patients developed small groin nodes on the side of the injection and in one of these the nodes were tender to palpation. The intradermal skin tests were negative in all cases.

### DISCUSSION

The results reported in both groups of patients in this study are in agreement with general experience in treating glioblastoma multiforme by conventional methods. It is clear that injected irradiated autologous tumour cells have not improved the survival of patients treated by surgery and radiotherapy. Patients who received irradiated tumour cells were all dead by 30 months whereas 5 (14%) of the control group survived for more than 3 years, one patient still being alive at 72 months. The initial mortality was equally rapid in both groups.

When the results are analysed according to sex there appears to be a transient advantage for female patients in both treatment groups. In laboratory experiments differing responses have been observed in male and female rats bearing chemically induced gliomata, suggesting that these tumours may be influenced by hormonal factors (Hopewell and Wright, 1969). The male predominance in human gliomata and the fact that male patients tend to die earlier than female patients (Penman and Smith, 1954) adds support to this possibility.

Since the work of Foley (1953) and Prehn and Main (1957), who demonstrated tumour specific antigens in methylcholanthrene induced murine sarcomata, it has become clear that a host response specifically retarding neoplastic cell growth can be mounted in a variety of viral and chemically induced animal tumours as well as in some spontaneous tumours of experimental animals (Old and Boyse, 1964; Hammond, Fisher and Rolley, 1967). Sensitized lymphocytes or antibodies which react with autologous tumour cells in man have been demonstrated in a number of tumours including Burkitt's lymphoma (Klein *et al.*, 1967), melanoma (Lewis *et al.*, 1969), soft tissue sarcomata (Morton *et al.*, 1968), neuroblastoma (Hellström *et al.*, 1968) and colonic neoplasms (Gold, 1967).

An antibody (Ikonopisov *et al.*, 1970) and lymphocyte (Currie, Lejeune and Hamilton Fairley, 1971) response against tumour cells can be demonstrated after injection of irradiated autologous tumour cells in patients with malignant melanoma, but unfortunately this is not associated with tumour regression. Although immune lymphoid cells taken from patients or animals with growing tumours may react against the corresponding tumour cells *in vitro*, serum factors exist *in vivo* which block the action of immune lymphoid cells or tumour antibodies in the host with cancer (Hellström and Hellström, 1970; Currie and Basham, 1972).

A recent histological study has reported lymphocytic infiltration in 27 of 77 patients (35%) with Grades III or IV astrocytomata (Ridley and Cavanagh, 1971). This tissue cellular response may represent the morphological features of a host defence mechanism similar to that seen in other human tumours such as breast cancer (Hamlin, 1968; Bloom, Richardson and Field, 1970), neuroblastoma (Martin and Beckwith, 1968), seminoma (Dayan, 1966), and stomach cancer (Black, Opler and Speer, 1956). Levy, Mahaley and Day (1972) have reported that patients with glioblastomata and other intracranial neoplasms possessed peripheral blood lymphocytes which were specifically cytotoxic to cultured autologous tumour cells *in vitro*. They concluded that both anaplastic and well-differentiated tumours of the central nervous system can induce a tumour-specific, cell-mediated immune response in the host. However, previous attempts by this group to develop tumour specific antisera for treatment of glioblastoma were unsuccessful (Mahaley and Day, 1965; Mahaley, 1968, 1971). On the other hand, precipitation between glioblastoma extract and the patient's serum after, but not before, immunization with autologous tumour extract has been reported by Trouillas (1971). These findings have not been supported by the work of Delpech *et al.* (1972) who were unable to demonstrate anti-tumour antibody in glioblastoma. In a recent study, Lim and Kluskens (1972) compared antigens from a rat astrocytoma with those from normal rat brain and liver using immunodiffusion and immunoelectrophoresis and reported the presence of antigens that appeared to be specific for the tumour cells. Clearly, further studies are required to establish the presence of tumour associated antigen in human gliomata.

Immunological investigations of malignant gliomata following the administration of irradiated autologous cells must take into consideration the antigenicity of normal brain tissue which may be included in the injected material, as well as the influence which the blood–brain barrier might exert upon cellular and humoral immune mechanisms. In recent years, several normal brain-specific antigens have been identified and partially characterized although their precise role has not been elucidated (Hatcher and MacPherson, 1970). It is known that under certain conditions animals may react immunologically with their own brain tissue and cause an allergic encephalomyelitis (Paterson, 1966). Although median survival in our patients was relatively short, there was no evidence for a superimposed acute encephalomyelitis. Under normal conditions proteins cannot cross the blood–brain barrier but the presence of tumour, a surgical procedure, and perhaps also cerebral irradiation are all situations in which the blood–brain barrier may no longer remain intact and consequently humoral immune mechanisms may be able to operate more readily. On the other hand, if cellular immune mechanisms are implicated chiefly in immunological tumour rejection processes and this seems likely (Alexander and Hamilton Fairley, 1967), then even an intact blood–brain barrier would not be expected to prevent the passage of sensitized lymphocytes which are known to be capable of migrating through normal vascular endothelium (Gowans, 1959).

It is possible that immunological mechanisms operate to prevent metastases developing outside the central nervous system. Extra-neurogenic spread from astrocytomata is an exceedingly rare occurrence but it is clear from autografting of viable tumour tissue (Bloom *et al.*, 1960; Grace *et al.*, 1961) that typical gliomata can in fact grow at peripheral implantation sites. The occasional occurrence of rapidly growing extracranial deposits in patients with ventriculo-pleural or ventriculo-peritoneal shunts (Wolf, Cowen and Stewart, 1954; Wakamatsu *et al.*, 1971) suggests that the paucity of metastases in glioblastoma is due to mechanical rather than immunological factors.

Bearing in mind the remarkable mobility of the lymphocyte, the mere confinement of cerebral tumours within the central nervous system would not, *per se*, prevent an immunological reaction from occurring across the blood–brain barrier. Scheinberg and Taylor (1968) have demonstrated that rejection of an intracerebral graft of chemically induced mouse glioblastoma can be achieved by immunizing the animal with tumour cells and Freund's adjuvant. On the other hand, human glioblastomata are usually large and invariably incompletely excised, infiltrating widely and diffusely into brain substance, and it is likely that any immunological reaction, either natural or induced, would be inadequate to control residual tumour growth. Even if specific tumour antigenicity is present, excessive antigen production from a large bulk of tumour might exhaust or paralyse the host's immunological defences (Currie and Basham, 1972). On the other hand, specific antigenic material may in fact be lost from gliomata of high grade malignancy compared with relatively benign tumours of neural origin (Wickremesinghe and Yates, 1971), although this concept is not supported by the recent work of Levy *et al.* (1972) who found sensitized peripheral lymphocytes in patients with anaplastic and also well-differentiated intracranial tumours.

In the 10 patients of the present series who had multiple injections of irradiated autologous tumour cells, none showed positive intradermal skin tests to indicate the development of a cell-mediated local reaction against the injected tumour. On the other hand, in 6 glioblastoma patients, autografted with *viable* tumour cells by Grace *et al.* (1961), 2 of the 4 patients who rejected their autografts gave strongly positive wheal reactions with surrounding flair on skin testing several months later, whereas 2 patients who developed typical glioblastoma in their subcutaneous tissue did not. This suggests that a delayed hypersensitivity cell-mediated type of immunological reaction had developed against the antigens of the grafted cells. It was impossible to say whether these antigens were tumour associated or merely carried by normal brain tissue included in the injected material.

In the present study, evidence of the presence of a cellular immune reaction occurring within inguinal nodes draining the inoculation site was crudely sought by palpation but small tender groin nodes appeared in only one patient. More recently Anderson *et al.* (1970) have studied the histological appearance of the draining lymph nodes following the injection of irradiated autologous tumour cells in 8 patients with carcinoma of the breast and one patient with carcinoma of the colon. The findings were compared with the appearance of nodes removed from normal controls, subjects who had typhoid, paratyphoid A and B vaccine injections or skin allografts and one patient with varicose eczema. Two of the 9 patients with cancer showed large numbers of pyroninophilic blast cells in the nodes. Similar changes were seen in the 4 skin allograft recipients and the patient with varicose eczema; in all the remaining patients the nodes were normal.

Finally, it is necessary to consider the possible dangers which may be associated with attempts at immunotherapy, such as enhancement of tumour growth which has been encountered in animal experiments (Vaage, 1971; Law *et al.*, 1971) reported after the conclusion of this trial. In most cases in the present study there was good clinical evidence of tumour recurrence and this was proved histologically in 9 of the 10 patients subjected to autopsy. Although the precise cause of death in the remaining patient (E.B., 066110, see above) remains obscure, it should be noted that this patient did *not* receive autologous tumour cells but was treated with surgery and irradiation alone.

Present methods of treatment generally fail to achieve more than temporary growth restraint in the great majority of patients with glioblastoma multiforme. Radical surgery and post-operative irradiation are capable of eradicating the tumour in only a very small proportion of patients, but are often valuable in palliation. Against this background of therapeutic failure it is relevant to consider ways in which the management of this tumour can be made more effective.

Extensive necrotic areas in the tumour are frequently present and it is probable that foci of anoxic malignant cells account, at least in part, for the failure of radiotherapy to achieve more than temporary tumour control. Although the clinical history in patients with glioblastoma may be short, tumour growth rate is not necessarily rapid. The tumour mass is often large and relatively small changes in volume within a confined space or involving important cerebral areas are likely to be critical in producing functional disturbance. Johnson *et al* (1960) found that only 0·6% of glioblastoma cells were synthesizing DNA, indicating that the proliferating fraction of cells at any given time was very small. This is consistent with the presence of a high proportion of hypoxic non-proliferating cells. Radiotherapy under hyperbaric oxygen may therefore on theoretical grounds offer some advantages, but so far there has been little enthusiasm for treating gliomata in this way.

Glioblastoma is a particularly suitable tumour for assessing the possible advantages of fast neutron therapy, since the biological effect with this treatment is less dependent upon cellular oxygenation than with photon irradiation. So far, chemotherapy has had little to offer for this tumour. A trial of BCNU or CCNU, alkylating agents which pass the blood–brain barrier, has proved disappointing in recurrent cases (Wilson and Hoshino, 1969). The use of cytotoxic agents, either before or together with cerebral irradiation, has theoretical advantages which also need to be explored. In a recent randomized trial, however, a combination of irradiation and 5-fluorouracil had no advantage over irradiation alone for patients with glioblastoma (Edland, Javid and Ansfield, 1971). Until more effective cytotoxic or radiosensitizing agents against these resistant tumours are found there seems little hope of improving the prognosis with adjuvant chemotherapy.

The concept of clinical immunotherapy is at present based more on speculation than fact. Further fundamental research is needed in this field but should only be undertaken with caution and in collaboration with those experienced in immunological work.

Of the therapeutic options available to us at the present time, we believe that neutron therapy for glioblastoma would be worth exploring and a clinical trial to compare this treatment with super-voltage x-irradiation is now being planned in association with Dr Mary Catterall at Hammersmith Hospital.

APPENDIX

*Details of statistical evaluation*

The survival times of individual patients included in the trial are shown in Fig. 9 using a logarithmic scale. These are arranged in 8 groups derived from the possible combinations of grade, sex and whether or not the patient received "immunotherapy". Nine patients who did not survive to have radiotherapy or who received their radiotherapy at hospitals other than the Royal Marsden Hospital have been omitted; this is justified since the surgery and radiotherapy were identical whether or not the patient was selected to receive "immunotherapy."

The use of the logarithmic transformation in carrying out statistical tests of significance is appropriate where, as in the case of survival times, the distribution of values tends to be positively skew. Fig. 9

FIG. 9.—Individual survival times plotted on a logarithmic scale by sex, grade of tumour and treatment group.

also shows for each group a measure of location obtained by taking the antilog of the mean log value of each group. If the distribution of survival times is log normal, these measures of location can be interpreted as estimates of the population median, *i.e.* the time by which 50% of patients will have died. A 90% confidence interval for these values is also shown.

The variation within the 8 groups was rather less in the case of patients with Grade III tumours receiving " immunotherapy ", but the differences were not significant so that a pooled " within group " variance was used ($S^2 = 0{\cdot}1322$; $S = 0{\cdot}3635$; degrees of freedom = 54). The $t$-tests carried out were

" Immunotherapy " *v* Controls
for 4 combinations of sex and grade.

Grade III *v* Grade IV
for 4 combinations of sex and treatment.

Male *v* Female
for 4 combinations of grade and treatment.

For Grade III tumours females appeared to fare slightly better than males but the difference was far from significant. The survival times for Grade III tumours were generally longer than for Grade IV tumours but even in this case differences between corresponding subgroups were not significant, largely because of the paucity of Grade IV tumours in the trial (there were for example only one Grade IV female control and only 2 Grade IV females receiving " immunotherapy ". The only difference approaching significance was Grade III *v* Grade IV for males receiving " immunotherapy " ($t = 1{\cdot}718$; d.f. = 54; $P$ (one tail) $< 1/20$).

Tests of the differences between survival times for control and " immunotherapy " patients were also carried out with the sexes combined; these again were not significant. The relevant statistics were as follows:

*Grade III*

Controls:

mean ($\log_{10}t$) = 1·0350) S = 0·3490

" Immunotherapy ":

mean ($\log_{10}t$) = 1·0578) (47 d.f.)

difference = 0·0228

$t$ = 0·225; not significant.

*Grade IV*

Controls:

mean ($\log_{10}t$) = 0·9214) S = 0·3834

" Immunotherapy ":

mean ($\log_{10}t$) = 0·7218) (11 d.f.)

difference = 0·1996

$t$ = 0·934; not significant.

REFERENCES

ALEXANDER, P. & HAMILTON FAIRLEY, G. (1967) Cellular Resistance to Tumours. *Br. med. Bull.*, **23**, 86.

ANDERSON, J. M., DESOUSA, M. A., HALNAN, K. E., KELLY, F. & HANNAH, G. (1970) Assessment of Immunization by Autotransplantation of Human Cancer. *Br. J. Surg.*, **57**, 557.

BLACK, M. M., OPLER, S. R. & SPEER, F. D. (1956) Structural Representations of Tumor-Host Relationships in Gastric Carcinoma. *Surgery, Gynec. Obstet.*, **102**, 599.

BLOCH, M., BLOOM, H. J. G., PENMAN, J. & WALSH, L. (1966) Observations on Patients with Cerebral Astrocytoma (Glioblastoma Multiforme) Treated by Irradiation under Whole-body Hypothermia. *Br. J. Cancer*, **20**, 722.

BLOOM, H. J. G., RICHARDSON, W. W. & FIELD, J. R. (1970) Host Resistance and Survival in Carcinoma of the Breast: a Study of 104 Cases of Medullary Carcinoma in a Series of 1411 Cases of Breast Cancer Followed for 20 Years. *Br. med. J.*, i, 181.

BLOOM, W. H., CARSTAIRS, K. C., CROMPTON, M. R. & MCKISSOCK, W. (1960) Autologous Glioma Transplantation. *Lancet*, ii, 77.

CHURCHILL-DAVIDSON, I. (1967) Therapeutic Uses of Hyperbaric Oxygen. *Ann. R. Coll. Surg.*, **39**, 164.

CRAIG, W. M., DODGE, H. W. JR. & SVIEN, H. J. (1957) Brain Tumours: Practical Approach and Classification. *Minn. Med.*, **40**, 471.

CURRIE, G. A. (1972) Eighty Years of Immunotherapy: a Review of Immunological Methods Used for the Treatment of Human Cancer. *Br. J. Cancer*, **26**, 141.

CURRIE, G. A. & BASHAM, C. (1972) Serum Mediated Inhibition of the Immunological Reactions of the Patient to His Own Tumour: a Possible Role for Circulating Antigen. *Br. J. Cancer*, **26**, 427.

CURRIE, G. A., LEJEUNE, F. & HAMILTON FAIRLEY, G. (1971) Immunization with Irradiated Tumour Cells and Specific Lymphocyte Cytotoxicity in Malignant Melanoma. *Br. med. J.*, ii, 305.

DAYAN, A. D. (1966) The Pathology of Testicular Tumours. *Hosp. Med.*, **1**, 126.

DELPECH, B., DELPECH, A., CLEMENT, J. & LAUMONIER, R. (1972) Étude Immunochimique et Immunologique des Tumeurs du Cerveau Humain. *Int. J. Cancer*, **9**, 374.

EARLE, K. M., RENTSCHLER, E. H. & SNODGRASS, S. R. (1957) Primary Intracranial Neoplasms; Prognosis and Classification of 513 Verified Cases. *J. Neuropath.*, **16**, 321.

EDLAND, R. W., JAVID, M. & ANSFIELD, F. J. (1971) Glioblastoma Multiforme. *Am. J. Roentg.*, **111**, 337.

FOLEY, E. J. (1953) Antigenic Properties of Methylcholanthrene-induced Tumors in Mice of the Strain of Origin. *Cancer Res.*, **13**, 835.

FREINKEL, S. & GORMAN, J. (1958) Glioblastoma Multiforme—Review of 219 Cases with Regard to Natural History, Pathology, Diagnostic Methods and Treatment. *J. Neurosurg.*, **15**, 489.

GOLD, P. (1967) Circulating Antibodies against Carcinoembryonic Antigens of the Human Digestive System. *Cancer, N.Y.*, **20**, 1663.

GOWANS, J. L. (1959) The Recirculation of Lymphocytes from Blood to Lymph in the Rat. *J. Physiol.*, **146**, 54.

GRACE, J. T., PERESE, D. M., METZHAR, R. S., SASABE, T. & HOLRIDGE, B. (1961) Tumor Autograft Responses in Patients with Glioblastoma Multiforme. *J. Neurosurg.*, **18**, 159.

HADDOW, A. & ALEXANDER, P. (1964) An Immunological Method of Increasing the Sensitivity of Primary Sarcomas to Local Irradiation with X-rays. *Lancet*, i, 452.

HAMILTON FAIRLEY, G. (1969) Immunity to Malignant Disease in Man. *Br. med. J.*, ii, 467.

HAMLIN, I. M. E. (1968) Possible Host Resistance in Carcinoma of Breast: a Histological Study. *Br. J. Cancer*, **22**, 383.

HAMMOND, W. G., FISHER, J. C & ROLLEY, R. J (1967) Tumor-specific Transplantation Immunity to Spontaneous Mouse Tumors. *Surgery, St. Louis*, **62**, 124.

HATCHER, V. B. & MACPHERSON, C. F. (1970) Studies on Brain Antigens. 3. Purification and Characterization of a Water-soluble Bovine Antigen Specific for Nervous Tissue (alpha-BASNT). *J. Immun.*, **104**, 633.

HELLSTRÖM, K. E. & HELSTROM, I. A. (1970) Immunological Enhancement as Studied by Cell Culture Technique. *A. Rev. Microbiol.*, **24**, 373.

HELLSTRÖM, K. E., HELLSTRÖM, I. A., PIERCE, G. E. & BILL, A. H. (1968) Demonstration of Cell-bound Humoral Immunity against Neuroblastoma Cells. *Proc. natn. Acad. Sci., U.S.A.*, **60**, 1231.

HITCHCOCK, E. & SATO, F. (1964) Treatment of Malignant Gliomata. *J. Neurosurg.*, **21**, 497.

HOPEWELL, J. W. & WRIGHT, E. A. (1969) The Importance of Implantation Site in Cerebral Carcinogenesis in Rats. *Cancer Res.*, **29**, 1927.

IKONOPISOV, R. L., LEWIS, M. G., HUNTER-CRAIG, I. D., BODENHAM, D. C., PHILLIPS, T. M., COOLING, C. I., PROCTOR, J., HAMILTON FAIRLEY, G. & ALEXANDER, P. (1970) Autoimmunization with Irradiated Tumour Cells in Human Malignant Melanoma. *Br. med. J.*, ii, 752.

JELSMA, R. & BUCY, P. C. (1969) Glioblastoma Multiforme: its Treatment and Some Factors Affecting Survival. *Archs Neurol., Chicago*, **20**, 161.

JOHNSON, H. A., HAYMAKER, W. E., RUBINI, J. R., FLIEDNER, T. M., BOND, V. P., CRONKITE, E. P. & HUGHES, W. L. (1960) A Radio-autographic Study of a Human Brain and Glioblastoma Multiforme after the *in vivo* Uptake of Tritiated Thymidine. *Cancer, N.Y.*, **13**, 636.

KERNOHAN, J. W. & SAYRE, G. P. (1952) Tumours of the Central Nervous System. *Atlas of Tumor Pathology*. Section 10, fasc. 35. Washington: A.F.I.P.

KLEIN, G., CLIFFORD, P., KLEIN, E., SMITH, R. T., MINOWADA, J., KOURILSKY, F. M. & BURCHENAL, J. H. (1967) Membrane Immunofluorescence Reactions of Burkitt Lymphoma Cells from Biopsy Specimens and Tissue Cultures. *J. natn. Cancer Inst.*, **39**, 1027.

LAW, L. W., APPELLA, E., WRIGHT, P. W. & STROBER, S. (1971) Immunologic Enhancement of Allogeneic Tumour Growth with Soluble Histocompatibility-2 Antigens. *Proc. natn. Acad. Sci., U.S.A.*, **68**, 3078.

LAWRENCE, J. H., TOBIAS, C. A., BORN, J. L., WANG, C. C. & LINFOOT, J. H. (1962) Heavy-particle Irradiation in Neoplastic and Neurologic Disease. *J. Neurosurg.*, **19**, 717.

LEVY, N. L., MAHALEY, M. S. JR. & DAY, E. D. (1972) *In vitro* Demonstration of Cell-mediated Immunity to Human Brain Tumors. *Cancer Res.*, **32**, 477.

LEWIS, M. G., IKONOPISOV, R. L., NAIRN, R. C., PHILLIPS, T. M., HAMILTON FAIRLEY, G., BODENHAM, D. C. & ALEXANDER, P. (1969) Tumour-specific Antibodies in Human Malignant Melanoma and their Relationship to the Extent of the Disease. *Br. med. J.*, iii, 547.

LIM, R. & KLUSKENS, L. (1972) Immunological Specificity of Astrocytoma Antigens. *Cancer Res.*, **32**, 1667.

MAHALEY, M. S. JR. (1968) Immunological Considerations and the Malignant Glioma Problem. *Clin. Neurosurg.*, **15**, 175.

MAHALEY, M. S. JR. (1971) Immunological Studies with Human Gliomas. *J. Neurosurg.*, **34**, 458.

MAHALEY, M. S. JR. & DAY, E. D. (1965) Immunological Studies of Human Gliomas. *J. Neurosurg.*, **23**, 363.

MARTIN, R. F. & BECKWITH, J. B. (1968) Lymphoid Infiltrates in Neuroblastomas: their Occurrence and Prognostic Significance. *J. Pediat. Surg.*, **3**, 161.

MATSUKADO, Y., McCARTHY, C. S. & KERNOHAN, J. W. (1961) The Growth of Glioblastoma Multiforme (Astrocytomas Grades III and IV) in Neurosurgical Practice. *J. Neurosurg.*, **18**, 636.

MORTON, D. L., MALMGREN, R. A., HOLMES, E. C. & KETCHAM, A. S. (1968) Demonstration of Antibodies against Human Malignant Melanoma by Immunofluorescence. *Surgery, St. Louis*, **64**, 233.

OLD, L. J. & BOYSE, E. A. (1964) Immunology of Experimental Tumors. *A. Rev. Med.*, **15**, 167.

PATERSON, P. Y. (1966) Experimental Allergic Encephalomyelitis and Autoimmune Disease. *Adv. Immunol.*, **5**, 131.

PENMAN, J. & SMITH, M. C. (1954) *Intracranial Gliomata.* Medical Research Council Special Report Series No. 284.

PREHN, R. T. & MAIN, J. M. (1957) Immunity to Methylcholanthrene-induced Sarcomas. *J. natn. Cancer Inst.*, **18**, 769.

RIDLEY, A. & CAVANAGH, J. B. (1971) Lymphocytic Infiltration in Gliomas: Evidence of Possible Host Resistance. *Brain*, **94**, 117.

ROTH, J. G. & ELVIDGE, A. R. (1960) Glioblastoma Multiforme: a Clinical Survey. *J. Neurosurg.*, **17**, 736.

SCHEINBERG, L. C. & TAYLOR, J. M. (1968) Immunological Aspects of Brain Tumours. *Prog. Neurol. Surg.*, **2**, 267.

SWEET, W. H., SOLOWAY, A. H. & BROWNELL, G. L. (1961) Boron Slow Neutron Capture Therapy of Operatively Exposed Gliomas. *II Int. Congr. Neurol. Surg.*, Washington D.C.

TAVERAS, J. M., THOMPSON, H. G. & POOL, J. L. (1962) Should we Treat Glioblastoma Multiforme? *Am. J. Roentg.*, **87**, 473.

TROUILLAS, P. (1971) Carcino-fetal Antigen in Glial Tumours. *Lancet*, ii, 552.

VAAGE, J. (1971) Concomitant Immunity and Specific Depression of Immunity by Residual or Reinjected Syngeneic Tumor Tissue. *Cancer Res.*, **31**, 1655.

WAKAMATSU, T., MATSUO, T., KAWANO, S., TERAMOTO, S. & MATSUMURA, H. (1971) Glioblastoma with Extracranial Metastasis through Ventriculopleural Shunt. Case Report. *J. Neurosurg.*, **34**, 697.

WICKREMESINGHE, H. R. & YATES, P. O. (1971) Immunological Properties of Neoplastic Neural Tissues. *Br. J. Cancer*, **25**, 711.

WILSON, C. B. & HOSHINO, T. (1969) Current Trends in the Chemotherapy of Brain Tumours with Special Reference to Glioblastomas. *J. Neurosurg.*, **31**, 589.

WOLF, A., COWEN, D. & STEWART, W. B. (1954) Glioblastoma with Extraneural Metastasis by Way of a Ventriculopleural Anastomosis. *Trans. Am. neurol. Ass.*, **79**, 140.